# Postural Coordination during Socio-motor Improvisation

**DOI:** 10.3389/fpsyg.2016.01168

**Published:** 2016-08-05

**Authors:** Mathieu Gueugnon, Robin N. Salesse, Alexandre Coste, Zhong Zhao, Benoît G. Bardy, Ludovic Marin

**Affiliations:** ^1^EuroMov Laboratory, University of MontpellierMontpellier, France; ^2^Institut Universitaire de FranceParis, France

**Keywords:** interpersonal coordination, posture, intrapersonal coordination, social interaction, mirror game, joint action

## Abstract

Human interaction often relies on socio-motor improvisation. Creating unprepared movements during social interaction is not a random process but relies on rules of synchronization. These situations do not only involve people to be coordinated, but also require the adjustment of their posture in order to maintain balance and support movements. The present study investigated posture in such a context. More precisely, we first evaluated the impact of amplitude and complexity of arm movements on posture in solo situation. Then, we assessed the impact of interpersonal coordination on posture using the mirror game in which dyads performed improvised and synchronized movements (i.e., duo situation). Posture was measured through ankle-hip coordination in medio-lateral and antero-posterior directions (ML and AP respectively). Our results revealed the spontaneous emergence of in-phase pattern in ML direction and antiphase pattern in AP direction for solo and duo situations. These two patterns respectively refer to the simultaneous flexion/extension of the ankles and the hips in the same or opposite direction. It suggests different functional roles of postural coordination patterns in each direction, with in-phase supporting task performance in ML (*dynamical stability*) and antiphase supporting postural control in AP (*mechanical stability*). Although amplitude of movement did not influence posture, movement complexity disturbed postural stability in both directions. Conversely, interpersonal coordination promoted postural stability in ML but not in AP direction. These results are discussed in terms of the difference in coupling strength between ankle-hip coordination and interpersonal coordination.

## Introduction

During social interaction in everyday life, people stand in front of each other, discuss and move together. Bodily coordination and postural mimicry appear spontaneously (LaFrance, 1982; Schmidt et al., 2014). Indeed, people constantly improvise by spontaneously creating and synchronizing speeches and movements to those of the interactant (Nachmanovitch, 1990; Sawyer, 2001; Shockley et al., 2009). Both the creativity of individuals’ movements and the interpersonal synchronization play a fundamental role in social interactions: they promote affiliation between interactants and witness the success of the interaction (Ekman and Friesen, 1969; Wing and Gould, 1979; Krauss et al., 1996; Goldin-Meadow, 1999; Schmidt and Richardson, 2008; Hove and Risen, 2009; Wiltermuth and Heath, 2009). This joint activity of creating behaviors together is called *socio-motor improvisation.* It is defined as the creative action without a script or anticipated preparation of two or more people involved in an interpersonal interaction (Noy et al., 2011; Gueugnon et al., 2016). These latter studies showed that people were able to create improvised arm movements while staying synchronized together. Such an improvisation situation not only involves the upper part of the body with the creation of synchronized movements of the arms, but it also requires the maintenance of balance with delicate coordination between the lower and upper parts of the body (Stoffregen and Riccio, 1988; Lee, 1989; Bardy, 2004). Although movement creativity and movement synchronization (of any body parts) are obviously present during social interaction, no study has examined their impact on posture. In the present article, we investigated postural coordination of each interactant during social interaction by using a socio-motor improvisation task.

Inspired by the dynamical systems approach (e.g., Kelso, 1995), *intrapersonal postural coordination* (IPC) has been used to characterize postural control as a function of the interaction between an individual and his/her surrounding environment. Using an auditory or a visual head-tracking task, previous studies have shown that posture is mostly organized in two spontaneous stable coordination patterns when computing the relative phase between ankle and hip angular motion: (i) an in-phase pattern (*Ø_h-a_* ≈ 0–20°) for low-frequency and/or small-amplitude movements and (ii) an antiphase pattern (*Ø_h-a_* ≈ 160–180°) for movements of high frequency and/or large amplitude (Bardy et al., 1999, 2002; Marin et al., 1999; Ferry et al., 2007; Stoffregen et al., 2009). These two patterns respectively refer to the simultaneous flexion/extension of the ankles and the hips in the same or opposite direction. The increase in movement frequency or amplitude to a critical value generally induces a loss of stability of the current pattern of coordination, thus yielding a transition from one pattern to the other one, accompanied by fluctuations in the transition region (Marin et al., 1999; Bardy et al., 2002; Varlet et al., 2011). These are fundamental studies revealing that stable IPC patterns emerge through the coalescence of external constraints and the intrinsic dynamics of the postural system (Bardy, 2004). However, IPC patterns were evaluated during tracking tasks requiring ample head oscillations, which are difficult to generalize to our daily social interactions. In addition, these studies involved laboratory-based supra-postural situations, which have now to be completed by the evaluation of IPCs in a more ecological context. Indeed in natural social interactions, people do not quiet stand in front of each other, but create and produce functional arm movements to accompany speech, pointing at an external object, or even touching each other. Posture is therefore constrained by the creation of these movements as well as by those of other persons. Thus, as movement coordination emerges between the interactants (Isenhower et al., 2010; Schmidt et al., 2014), they influence posture in return. In this current study, we focused on the impact of such social interaction on postural coordination.

Several studies have shown an effect of arm movements on posture. Abe and Yamada (2001) investigated the influence of simple oscillatory arms movements on postural coordination. By computing and analyzing the relative phase between various joints of the body (i.e., hip-shoulder and ankle-shoulder), they found that increasing the frequency of arm movements influenced postural stability. Others observed that a complex bi-manual coordination affected postural sway – resulting in an increase in sway activity – suggesting that performing complex arm movements disrupts postural control (Forner-Cordero et al., 2007). Additionally, Varlet et al. (2011) investigated the effect of interpersonal coordination on posture. Using the head-tracking task, they showed that spontaneous interpersonal coordination influenced the stability of IPC and constrained participants to switch from one pattern of coordination to another at the common frequency. However, their results did not reveal if this influence was positive (i.e., stabilized the posture and promoted its coordination) or negative (i.e., destabilized posture and hampered its coordination). Ramenzoni et al. (2011, 2012) investigated how intentional interpersonal end-effector coordination modulated posture by exploring torso movements. In this joint task, participants were assigned to different fine-tuned roles: One participant (the holder) held a target circle while the other participant (the pointer) had to place a pointer inside the circle without touching it. They showed not only that this joint task impacted the way each actor coordinated movements of hand and torso, but also that the role performed during the interaction (i.e., holder vs. pointer) modulated postural sway. Taken together, these studies suggest that arm movements and interpersonal coordination influence postural control. Finally, recent studies proposed the mirror game paradigm to investigate in more details these two parameters of social interaction (Hart et al., 2014; Noy et al., 2011, 2015; Gueugnon et al., 2016; Slowinski et al., 2016). In this game, two participants were asked to move a handle in the medio-lateral (ML) direction by creating interesting, various and complex movements while staying as synchronized as possible. Movement creativity was observed through movement complexity in terms of frequency, and amplitude (i.e., multi-frequency and multi-amplitude movements; Gueugnon et al., 2016). As in Ramenzoni et al.’s (2011, 2012) studies, each participant had a specific role: leader and follower. These roles allowed the manipulation of interpersonal coordination, as the follower had to track and imitate the leader’s movements.

The aim of this study was to investigate the postural organization underlying social interactions (i.e., socio-motor improvisation). More precisely, we evaluated two important parameters of social interaction on posture: creativity of end-effectors movements and interpersonal coordination. In order to distinguish these two effects, we first manipulated the creativity of the end-effector movements (amplitude and complexity) without interpersonal end-effector coordination (i.e., Solo situation). Once the effect of creativity is revealed, we evaluated in the second part the influence of interpersonal coordination on IPC when pairs of participants performed synchronized and improvised arm movements in a leader-follower situation of the mirror game task (i.e., Duo situation). To obtain a global overview of the postural coordination, we assessed postural coordination in the ML instructed direction of arm movements. But we also investigated the antero-posterior (AP; uninstructed) direction based on previous study that demonstrated the emergence of postural coordination in both directions (Zhang et al., 2007). Ramenzoni et al. (2012) have for instance analyzed AP and ML movements to obtain a global overview of the postural movements.

Two main hypotheses were made. (i) Based on the first part, we expected that performing more complex and larger arm movements would influence IPC by decreasing its stability (either by switching from one pattern to another or by lowering the stability of these patterns) compared to performing simpler and smaller movements. (ii) We predicted that interpersonal coordination would promote postural stability in the duo situation. Moreover, by comparing Leader and Follower conditions, we expected to observe a difference between the two roles, with the follower being more influenced by the social interaction than the leader (since he had to follow the leader’s movements).

## Materials and Methods

### Participants

Ninety six healthy subjects, from the Department of Sport Sciences at Montpellier University, were recruited for the experiment, constituting 48 dyads of participants. Within each pair, participants did not know each other. This experiment was part of a larger study in which each dyad performed several steps and motor tasks. In the current experiment, we focused on the first two steps: a Solo Imposed task and a Duo Improvisation task. For a more detailed description of the entire study, the reader can refer to Gueugnon et al. (2016).

Of these 48 pairs, 42 dyads were included in the Solo situation (with 20 male dyads; mean age 20.2 years *±* 2.6, mean height 1.71 m ± 0.11, and mean weight 63.6 kg *±* 13.9), but only 27 dyads (with 10 male dyads; mean age 20.1 years ± 2.1, mean height 1.68 m ± 0.10, and mean weight 59.4 kg ± 10.52) were retained for both Solo and Duo situations (some dyads were excluded for technical reasons, see *Data Analysis and Dependent Variables*). All subjects had normal or corrected-to-normal vision and were right-handed according to the Edinburgh Handedness Inventory. Written consent forms were obtained by following the Montpellier University guidelines. The entire experiment conformed the Declaration of Helsinki and the regulatory standards given by the EuroMov Ethical Committee.

### Task and Procedure

**FIGURE 1:**
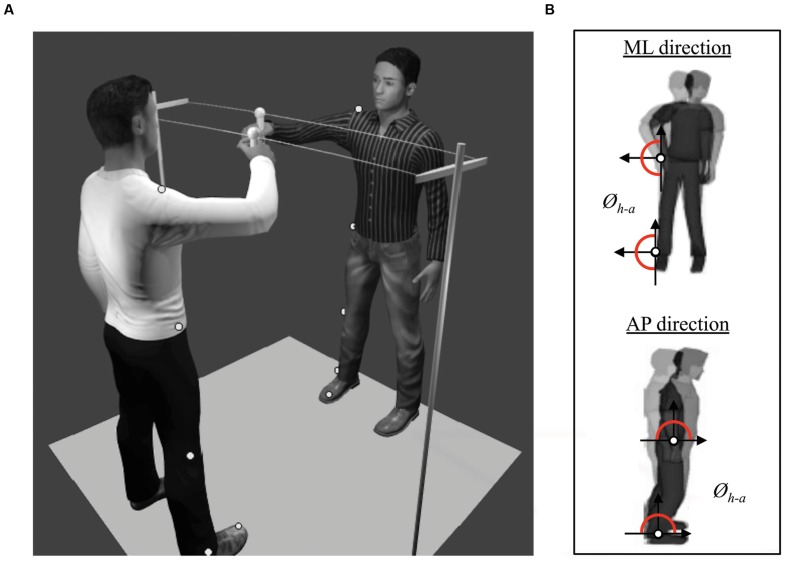
**(A)** Set-up of the protocol showing two participants engaged in a one-dimensional motor improvisation task (a.k.a. mirror game) consisting in sliding a hand-held handle along a string (Duo situation). Five passive markers were pasted on each participant to record the angular displacements of relevant joints. The “MakeHuman” model is part of the open source modeling tool “MakeHuman” (www.makehuman.org). Blender 2.72b was used to create the environmetal set-up. **(B)** Illustration of the computed relative phase (*Ø_h-a_*) between the angular position of hip and ankle in ML and AP directions (respectively top and bottom).

Participants of each dyad stood up in front of each other at a distance of 1 m. They held a handle with their right hand. The two handles, placed at a distance of 0.45 m from each other, were attached to a 1.8 m-long string. The string was horizontally positioned at around the shoulder’s level. Participants had to move the handle in the ML direction (**Figure 1A**).

**FIGURE 2:**
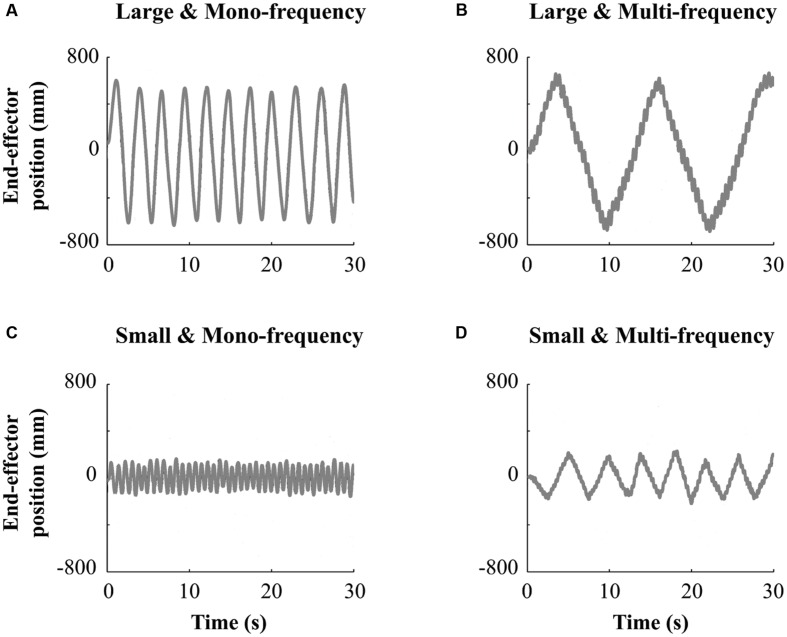
**Representative temporal series obtained in the Solo situation for one subject for the Large and Mono-frequency group **(A)**; Large and Multi-frequency group **(B)**; Small and Mono-frequency group **(C)** and Small and Multi-frequency group **(D)****.

In the Solo situation, participants were randomly split in four different groups to manipulate the creativity of arm movements in terms of movement complexity and amplitude. A two-by-two (between group) experimental design was used. Movement complexity was manipulated by asking participants to either “produce one simple oscillation at their preferential frequency” (i.e., Mono-frequency group with *N* = 42 participants) or to “perform a double oscillation, superimposing a faster frequency component onto their natural oscillation” (i.e., Multi-frequency group with *N* = 42 participants) yielding more complex arm movements for this last group. Movement amplitude was manipulated by instructing participants from the small amplitude group (i.e., Small group with *N* = 23 participants) to “perform movements with an amplitude smaller than their shoulder width.” Participants from the large amplitude group (i.e., Large group with *N* = 61 participants) were instructed to “perform movements with an amplitude larger than their shoulder width.” Experimenters first gave the corresponding instruction and briefly demonstrated the type of movement each participant had to perform. Finally, each participant performed alone one trial of 30 s facing the other immobile participant (one after the other). **Figure 2** summarizes the movement requested in each group.

In the Duo situation, all 27 dyads of participants from all groups performed the mirror game. Subjects playing together were instructed to “create interesting, complex and various movements and stay as coordinated as possible” by moving the handle they were holding along the string (see **Figure 1A**). One participant, designated as the leader, was improvising, whereas the second participant followed the leader’s movements. Each participant performed one trial of 45 s as Leader and another 45-s trial as Follower. In a recent study, we found that this duration was sufficient to capture the specificity of such movements (Slowinski et al., 2016).

### Data Analysis and Dependent Variables

**FIGURE 3:**
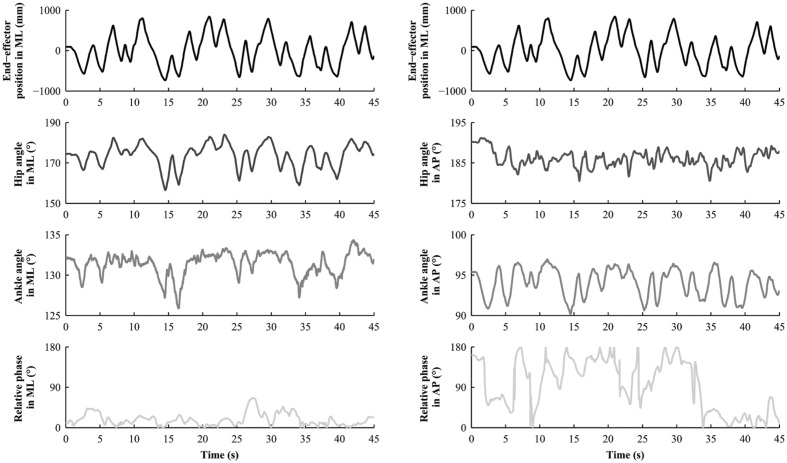
**Representative temporal series obtained in the Duo situation for one subject (Leader condition)**.

Movements of each participant were captured at a sampling frequency of 100 Hz by eight infrared cameras (Nexus MX13 Vicon System©), tracking six reflecting markers: one at the top of each handle to capture the movement of the end-effector; and five on the right side of the body of each subject to capture postural motion (see **Figure 1A**). These five markers were located on the shoulder (acromion), hip (greater trochanter), knee (estimated knee joint center), ankle (lateral malleolus) and toe (head of the first metatarsal), and were used to compute the angular motion of the hip and the ankle (Varlet et al., 2011). Therefore, the displacements of each marker in the AP and in the ML directions were extracted, centered around zero and low-pass filtered using a second order dual-pass Butterworth filter with a cut-off frequency of 7.5 Hz. Unfortunately, some markers were occasionally masked or shadowed, and disappeared from the cameras’ field of view, which is not uncommon during postural recordings. In order to adopt the same procedure for all subjects, we removed the corresponding dyad if one marker was missing. In total, 21 over 96 subjects were excluded because one or two markers (out of the six) were missing. It represents an exclusion of less than 5% of the totality of the markers. Therefore, we excluded 21 over 96 participants (22%) corresponding respectively to 27 dyads over 48. For all remaining dyads, we calculated the angular position of the hips and the ankles in AP and ML directions. An example of the time series is illustrated in **Figure 3** for Duo situation. From these angular positions, two dependent variables were computed to measure IPC.

We first calculated and averaged the cross-spectral coherence (CSC) at the peak frequency of hip and ankle angular positions. CSC quantifies the correlation between the two signals at the maximal peak frequency. The higher the correlation, the stronger the coupling between the two signals (Richardson et al., 2005; Schmidt et al., 2007). A CSC value of 1 indicates that the two signals are strongly coordinated at the same dominant frequency, and a CSC of 0 indicates no common dominant frequency hence no coordination. CSC was recently used to investigate ankle-hip coordination (Sofianidis et al., 2015). Second, to evaluate the dynamical coordination between these two joints, we computed the ankle-hip relative phase (**Figure 1B**) using the cross-wavelet transform (Varlet et al., 2011; Schmidt et al., 2014; Issartel et al., 2015). Indeed, compared to classical methods to compute relative phase, the cross-wavelet transform is adapted to complex time series with non-stationary properties, such as the time series obtained in the current study. It evaluates the cross-spectrum of two signals as a function of time, and informs about the relative phase patterns between signals for different frequencies and at each point in time (Torrence and Compo, 1998; Issartel et al., 2006; Issartel et al., 2015). In our analyses, all cross-wavelet transforms were performed using the complex Morlet wavelet of order 8 and a band of frequencies ranging from 0.10 to 2 Hz, which captures time scales in the time series’ activity. In order to obtain a global view of the behavioral coordination, we averaged the relative phases contained in the range of frequencies for each time sample and obtained a value of relative phase between 0° and 180° at each point in time (i.e., instantaneous relative phase). Because spontaneous IPC appears intermittently, the standard deviation of the relative phase is not appropriate to assess the stability of this coordination. Hence, we computed the distribution of the ankle-hip relative phase in order to obtain the pattern(s) of coordination involved and its stability (Richardson et al., 2007; Varlet et al., 2011). In line with the literature, nine 20° regions of relative phase from 0° to 180° and the percentage of occurrence in each of these regions were consequently calculated. In our study, the in-phase pattern (*Ø_h-a_*≈ 0–0°) corresponded to a simultaneous flexion or extension of the ankles and the hips (in the AP and in the ML plane), and the antiphase pattern (*Ø_h-a_*≈ 160–180°) referred to a flexion or extension of the ankles and the hips in the opposite direction. The higher the occurrence of the stable pattern, the more stable the coordination.

Finally, we also controlled the amplitude and the complexity of the end-effector movements in Solo and Duo situations. Using the displacement of the marker placed at the top of the handles, we first computed the amplitude difference in each signal for all participants. Second, to verify the complexity of the movements, we computed the width of the spectrum of frequencies significantly present in the signal. To do so, we used a wavelet transform analysis on the end-effector position in the instructed direction. This method provides a decomposition of the signal in the time-frequency space and shows the significant frequencies present in the signal at each moment of time (Issartel et al., 2006, 2015). The complex Morlet wavelet of order 8 was used for this analysis. In order to compare end-effectors movements in Solo and in Duo situations, we selected a common band of frequencies from 0.25 to 5 Hz that encompassed end-effector movement frequencies for both situations (Issartel et al., 2006, 2015). This variable represents the amount of significant frequencies present in this window. We computed and averaged the sum of the frequencies significantly present in this band of frequencies. Then, we divided this value by the maximal number of elements present in the window to obtain a value comprised between 0 and 1. The higher the value, the higher the complexity since the signal contained more significant frequencies.

### Statistical Analysis

In order to investigate the effect of arm movements on IPC independently of the social interaction, we analyzed IPC in the Solo situation alone. As dyad groups had different sizes, non-parametric tests were performed, using the Mann–Whitney test to compare (i) Small and Large groups and (ii) Mono-frequency and Multi-frequency groups. When necessary, we used the Friedman ANOVA and the Wilcoxon sign rank test to distinguish the differences. Effects sizes were, when possible, reported using *r* where a small effect is 0.1, a medium effect is 0.3 and a large effect is 0.5 (Coolican, 2009).

To explore the influence of interpersonal end-effector coordination on IPC, we then compared IPC in the Solo situation and in the Duo situation. To do so, repeated-measures ANOVAs were run on our dependent variables. If necessary, Greenhouse–Geisser adjustments were made for violations of sphericity, and Newman–Keuls *post hoc* tests were computed when necessary. Effects sizes of the repeated-measures ANOVA design are reported below in the results section using the partial eta squared $\eta_{p}^{2}$ (Bakeman, 2005) interpreted according to Cohen’s (1988) article , where 0.02 corresponds to a small effect, 0.13 to a medium effect and 0.26 to a large effect. In this experiment, we looked for effects between Duo and Solo situations and also between Leader and Follower conditions.

## Results

In this section, we first verify the effect of arm movements on IPC without social interaction (i.e., in Solo situation only). We then focused on the effect of interpersonal end-effector coordination on IPC in Part II, before discussing all results. In both parts I and II, we first analyzed end-effector movements in the ML direction, and then postural coordination in ML and AP directions separately.

### Part I

#### End-Effector Movements in ML Direction

As anticipated, the Mann–Whitney analysis on the amplitude difference revealed that this variable was significantly higher for the Large group (1.15 m ± 0.22) than for the Small group (0.40 m ± 0.12; *U* = 0, *p* < 0.001, *r* = 0.77).

For the width of the spectrum, our statistical analysis showed a significant difference between Mono- and the Multi-Frequency groups (0.28 ± 0.18 and 0.48 ± 0.18 respectively; *U* = 405, *p* < 0.001, *r* = 0.47). Both results confirmed that participants followed the instructions: (i) Large and Small groups were well different in terms of amplitude of the end-effector movements and (ii) Mono-frequency and Multi-frequency groups were also significantly different in terms of the complexity of end-effector movements.

#### Postural Coordination in ML Direction

For the averaged CSC, the Mann–Whitney analysis revealed that the coordination between hips and ankles was greater for the Mono-frequency group (0.61 ± 0.31) than for the Multi-frequency group (0.36 ± 0.26; *U* = 489, *p* < 0.001, *r* = 0.54). No difference was observed between Small and Large groups (0.58 ± 0.30 and 0.45 ± 0.31 respectively; *p* = 0.053).

**FIGURE 4:**
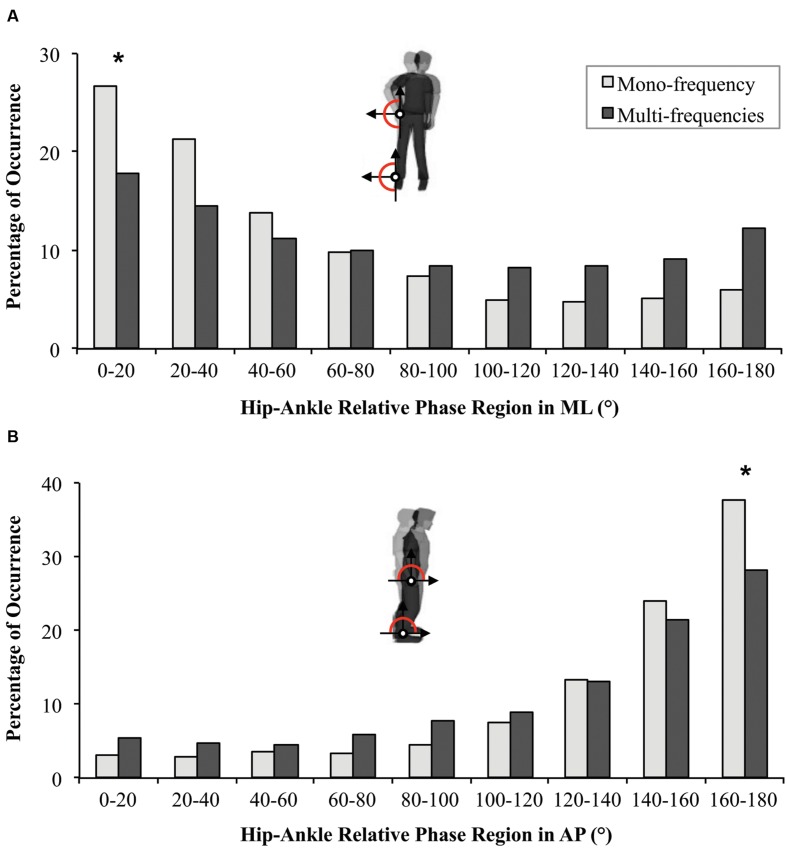
**Distribution of relative phase (*Ø_h-a_*) in ML **(A)** and AP directions **(B)** during Solo situations for participants from the Mono-frequency group (light gray) and from the Multi-frequency group (dark gray).** Statistical differences between the two groups are indicated by ^∗^.

For the distribution of the relative phase between 0 and 180°, a high occurrence of in-phase pattern was observed for the Small group (χ^2^ = 15.5, df = 8, *p* = 0.049), for the Large group (χ^2^ = 63.2, df = 8, *p* < 0.001) and for the Mono-frequency group (χ^2^ = 90.8, df = 8, *p* < 0.001), but not for the Multi-frequency group (*p* = 0.401). The comparisons between groups revealed (i) a greater occurrence of the in-phase pattern for the Mono-frequency group compared to the Multi-frequency group (*U* = 658, *p* = 0.045, *r* = 0.31; **Figure 4A**), and (ii) no differences between Small and Large groups (*p* = 0.737).

#### Postural Coordination in AP Direction

For CSC, the same results as the ones present in ML were observed revealing that coordination between hips and ankles was greater for the Mono-frequency group than for the Multi-frequency group (0.60 ± 0.25 and 0.34 ± 0.25 respectively; *U* = 398, *p* < 0.001, *r* = 0.67). No difference was observed between Small and Large groups (0.53 ± 0.25 and 0.45 ± 0.30 respectively; *p* = 0.295).

For the occurrence of relative phase between 0 and 180°, a high percentage of occurrence of the antiphase pattern was observed for the four groups (Mono-frequency group: χ^2^ = 176.1, df = 8, *p* < 0.001; Multi-frequency group: χ^2^ = 102.7, df = 8, *p* < 0.001; Small group: χ^2^ = 82, df = 8, *p* < 0.001; Large group: χ^2^ = 188.4, df = 8, *p* < 0.001). The comparisons between groups revealed (i) a higher occurrence of the antiphase pattern for the Mono-frequency group compared to the Multi-frequency group (*U* = 648.5, *p* = 0.036, *r* = 0.32; **Figure 4B**) and (ii) no difference between Small and Large groups (*p* = 0.647).

In summary, these results show that ankle-hip coordination emerged spontaneously in the instructed ML as well as in the uninstructed AP direction in a stable pattern (in-phase and antiphase respectively). The complexity of arm movements, contrary to its amplitude, seems to disturb IPC by decreasing the occurrence of the stable patterns in both directions and even more in ML (since participants from the Multi-frequency group did not exhibit a stable IPC pattern).

### Part II

In this part, we focus on the effect of interpersonal end-effector coordination on IPC by comparing Solo, Leader and Follower conditions.

#### End-Effector Movements in ML Direction

Since no significant effect of amplitude of end-effector movements was observed in Part I, we only compared the complexity of these movements between Solo, Leader and Follower conditions. A repeated-measures ANOVA reveal a Condition effect [*F*(1.86,98.6) = 58.90, *p* < 0.001, $\eta_{p}^{2}$ = 0.53]. As expected, it shows that end-effector movements performed in the Duo situation (Leader and Follower) were significantly more complex than those performed in the Solo situation (0.58 ± 0.10; 0.58 ± 0.10 and 0.35 ± 0.16 respectively; *p* < 0.001 for both).

#### Postural Coordination in ML Direction

**FIGURE 5:**
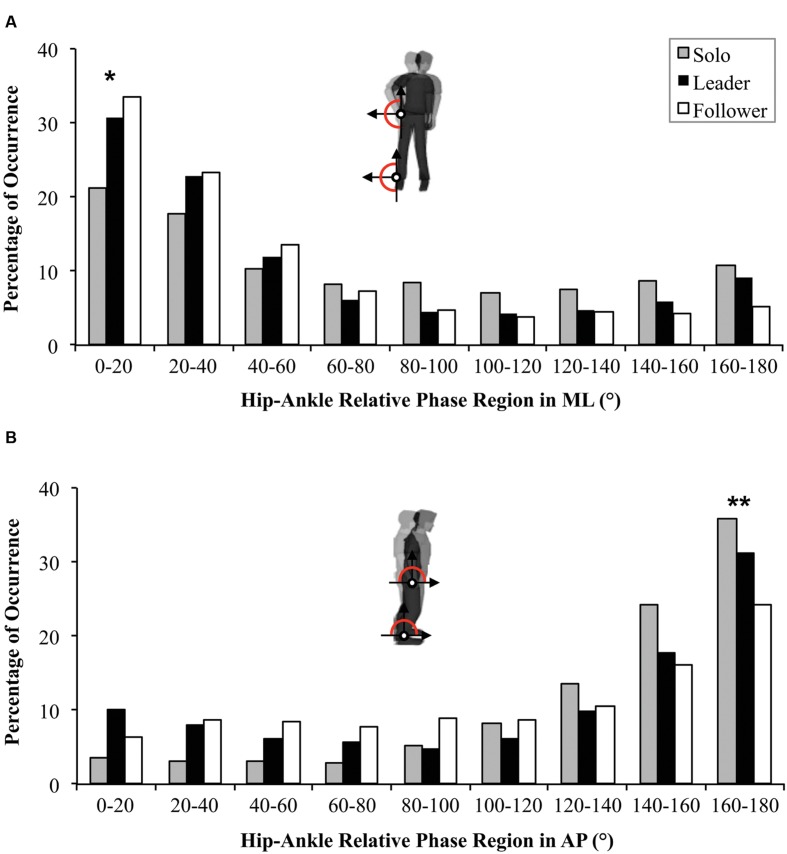
**Distribution of relative phase (*Ø_h-a_*) in ML **(A)** and in AP directions **(B)** between the Solo situation (gray) and Duo situations (Leader in black, Follower in white).** Statistical differences between Solo and Duo situations (both Leader and Follower) are indicated by ^∗^. Statistical differences between the three conditions (Solo, Leader and Follower) are indicated by ^∗∗^.

For CSC, a repeated-measures ANOVA failed to reveal a Condition effect between Solo, Leader and Follower conditions (0.50 ± 0.31; 0.41 ± 0.25 and 0.44 ± 0.25 respectively; *p* = 0.261). However, the repeated-measures ANOVA on the distribution of relative phase revealed a Region effect [*F*(1.88,99.6) = 58.7, *p* < 0.001, $\eta_{p}^{2}$ = 0.53] showing the general emergence of in-phase IPC (*p* < 0.001). A significant Region × Condition interaction [*F*(4.48,236.24) = 4.7, *p* < 0.001, $\eta_{p}^{2}$ = 0.08] revealed a greater occurrence of in-phase IPC for Leader and Follower conditions compared to the Solo condition (**Figure 5A**; *p* < 0.001 for both) but no difference between Leader and Follower conditions (*p* = 0.199).

#### Postural Coordination in AP Direction

For CSC, a repeated-measures ANOVA showed an effect of Condition [*F*(2,106) = 11.5, *p* < 0.001, $\eta_{p}^{2}$ = 0.18] revealing a greater correlation between hips and ankles for the Solo condition (0.50 *±* 0.29) than for Leader and Follower conditions (0.31 *±* 0.24 and 0.30 *±* 0.22 respectively; *p* < 0.001 for both comparisons).

For the distribution of the relative phase, the repeated-measures ANOVA showed a Region main effect [*F*(1.95,103.46) = 83.39, *p* < 0.001, $\eta_{p}^{2}$ = 0.60] confirmed by the general adoption of an antiphase IPC pattern (*p* < 0.001). A significant Region × Condition interaction [*F*(3.6,190.8) = 6.65, *p* < 0.001, $\eta_{p}^{2}$ = 0.11] showed that antiphase pattern was more adopted for Solo than for Leader and Follower conditions (*p* < 0.001 for both). The antiphase pattern was also significantly more present when the participant was leading than when he was following suggesting less postural stability for the follower (*p* < 0.001; **Figure 5B**).

To summarize, these results confirm the emergence of the in-phase pattern in ML, and of the antiphase pattern in AP. In addition, the comparison between Solo and Duo situations showed two different patterns of results in AP and ML. Interpersonal coordination seems to have improved IPC by increasing the occurrence of the stable pattern in ML. Conversely, it seems to have perturbed postural coordination in AP, but mostly for the follower participants.

## Discussion

The aim of the current experiment was to explore the postural organization underlying social interactions. First, we investigated the influence of arm movements on IPC without social interaction. More precisely, we addressed the question whether complexity and amplitude of arm movements would disturb IPC. Participants performed simple (i.e., mono-frequency) or complex (i.e., multi-frequency), and small or large amplitude, arm movements in the ML direction. We expected that performing more complex and larger movements would disturb postural coordination to a larger extent. Second, we examined the influence of interpersonal end-effector coordination on IPC when participants in a dyad performed synchronized improvised arm movements. Participants were asked to create interesting, various and complex movements while staying as synchronized as possible. One participant acted as the leader and the other one acted as the follower. We expected that interpersonal arm coordination would stabilize IPC compared to the Solo condition, and that acting as a follower – being more influenced by the other participant during the interaction – would exhibit higher IPC stability.

Our experiment revealed four main outcomes. (i) IPC emerged spontaneously in ML and in AP directions when performing arm movements in both Solo and Duo situations. (ii) Arm movement complexity, not amplitude, disturbed IPC in the Solo situation. (iii) Interpersonal end-effector coordination improved postural stability in the instructed ML direction but decreased it in the uninstructed direction. (iv) Acting as a follower decreased more the IPC stability in the uninstructed direction than acting as a leader.

### Emergence of Spontaneous IPC in Both Directions during Arm Movement

First, we observed a spontaneous emergence of stable postural coordination in the instructed as well as in the uninstructed arm movement direction (ML and AP respectively). The magnitude of the observed coherence (all means ranged between 0.30 and 0.61) are lower than those obtained by Sofianidis et al. (2015) in which participants were asked to follow an auditory stimulus yielding voluntary postural oscillations (coherence value around 0.8). However, they are comparable with those obtained in the literature for spontaneous interpersonal coordination (Schmidt and O’Brien, 1997; Richardson et al., 2005; Varlet et al., 2014; coherence value around 0.5 for these studies), suggesting a weak but persistent dynamical coupling with the emergence of relative coordination (Schmidt and Richardson, 2008). More precisely, postural coordination was captured by two stable patterns: an in-phase IPC in the instructed ML direction and an antiphase IPC in the uninstructed AP direction.

We believe that such a difference of pattern stability in both directions has its origin in two complementary but different types of stability: dynamical and mechanical stability. On the one hand, *dynamical stability* refers to the ability of the system to resist an external perturbation by maintaining the current pattern of coordination (e.g., Newell et al., 1993). A change from one stable pattern to another appears when the perturbation is too strong (Bardy et al., 2002). It is well known that the frequency of end-effector movements influences this stability. The in-phase postural pattern is indeed the most stable pattern underlying body movements performed at low frequency (c.f., Bardy et al., 2002). Abe and Yamada (2001) have also shown a change of coordination when the frequency of arm movements reached 0.79 Hz. In our study, the frequency of arm movement (Solo: 0.36 Hz; Leader: 0.28 Hz and Follower: 0.28 Hz) was lower than the critical value mentioned in the latter study that usually promotes in-phase coordination. This result is in line with the work from Zhang et al. (2007) who found that postural coordination is visible in ML during quiet stance (and for low frequencies). Our result extends this study in the context of supra-postural task in which the control of posture is not the main or only goal (Stoffregen et al., 1999). On the other hand, *mechanical stability* refers to the maintenance of equilibrium involving displacements of the center of mass and of the center of pressure. The antiphase pattern is the most mechanically stable pattern since it limits these displacements (e.g., Bonnet et al., 2011). In our situation, performing arm movements requires dynamical stability from the postural system in order to comply with the task instructions in ML. But at the same time, the postural system also necessitates to be mechanically stable in order to maintain a solid proximal support for performing end-effector movements. Our participants seem to have found the optimal balance between these two types of stability, by adopting the most efficient postural pattern (i) accompanying the end-effector movements in the instructed ML direction, while (ii) preserving the overall balance in the AP direction.

### Differential Adaptation of IPC to Movement Amplitude and Complexity

In the absence of interpersonal end-effector coordination (i.e., Solo situations), movement amplitude and complexity differently influenced IPC. First, performing large or small amplitude arm movements did not influence IPC, neither in terms of pattern nor in terms of stability in both ML and AP directions. This result is at first sight surprising since movement amplitude is known to affect postural coordination stability (Marin et al., 1999). Using the head-tracking task, Marin et al. (1999) found that amplitude of end-effector (head) movement equal to 0.23 m was sufficient to disturb IPC stability. In our situation, although the arm amplitude instruction seemed higher than 0.23 m (for Small group: 0.4 m ± 0.12; for Large group: 1.15 m ± 0.22), the shoulder/trunk displacements were in fact smaller than this value (0.04 m ± 0.02 and 0.19 m ± 0.11 respectively). These low displacements of the shoulder/trunk could explain why we did not observe any difference in IPC between Small and Large groups. Another possible explanation relates to a functional adaptation to the task. Participants performing large amplitude of arm movements could have redistributed their body weight from one foot to the other, accompanying arm movements from left to right, yielding in-phase postural oscillations in ML for large amplitudes. However, this alternative COP pattern (i.e., called “shifting”) has been only observed during prolonged quiet stance (e.g., Duarte and Zatsiorsky, 1999; Duarte et al., 2000).

Conversely and as expected, we observed that performing complex arm movements disturbed posture by decreasing the ankle-hip coordination and its stability (i.e., CSC and occurrence of the dominant stable pattern respectively), in both directions. This confirms and extends existing results in related bilateral situations (Forner-Cordero et al., 2007) showing that the stability of postural coordination is disturbed when performing unilateral complex end-effector movements. It also echoes recent findings on postural control at sea (e.g., Stoffregen et al., 2013). On a ship, the postural system is constrained by complex multi-frequency oscillations leading to a decrease in postural stability (with an increase in postural sway). We thus extend these results and show that performing multi-frequency arm movements also decrease postural stability. Finally, the loss of stable coordination patterns in ML direction for participants performing complex movements suggests that the deleterious effect of movement complexity is higher in the instructed direction. The movements being performed in the ML direction, their influence is more pronounced in this direction.

### Interpersonal Coordination Differently Influences Postural Stability in Both Directions

**FIGURE 6:**
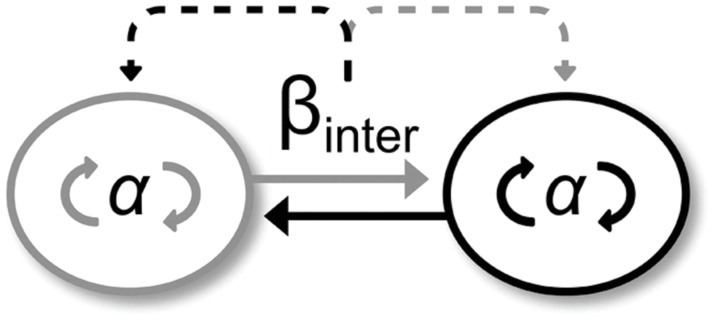
**A simple model capturing the difference in coupling strength between intra- and inter-personal levels, leading to the transfer from the stronger to the weaker.** The two circles represent the two participants interacting together (full line arrows) during an intentional interpersonal coordination task (strong coupling β_inter_) while maintaining their own postural coordination (weak coupling α). The coupling difference yields to a transfer from the stronger to the weaker type of coupling (dotted line arrows), leading to improvement in intrapersonal postural coordination.

As expected, participants performed more complex end-effector movements in Duo than in Solo situations (Duo: 0.58 ± 0.10; Solo Multi-frequency group: 0.42 ± 0.13 and Solo Mono-frequency group: 0.28 ± 0.16). However, the deleterious effect of arm movement complexity on IPC discussed above seems to have disappeared in the presence of interpersonal end-effector coordination in ML direction. On the contrary, we observed a higher occurrence of in-phase for Duo compared to the Solo situation, suggesting that interpersonal end-effector coordination positively impacted IPC. We believe that this result is related to the strength of interpersonal coordination. **Figure 6** illustrates our purpose. In our experiment, each participant (i.e., each circle) coordinated ankle-hip joints (i.e., α_intra_) to maintain balance and accompany movements, but also exchanged information leading to a coupling between their arm movements (i.e., β_inter_). When coordination is intentional, β_inter_ coupling is strong and witnesses an *absolute coordination*, whereas when coordination is spontaneous, β_inter_ coupling is weak, yielding to a *relative coordination* (e.g., Schmidt and Richardson, 2008). In Duo situations, participants intentionally coordinated their end-effector movements in the ML direction and spontaneously coordinated their ankle-hips joints yielding a strong interpersonal coupling (β_inter_) and a weak intrapersonal coupling (α_intra_). We believe that the strong interpersonal coupling (β_inter_) was transferred to the weak intrapersonal coupling (α_intra_) leading to a reinforcement of the intrapersonal coordination in ML. This result departs from that of Coey et al. (2011), who did not find an influence of spontaneous interpersonal coordination on the stability of intrapersonal bimanual coordination. Varlet et al. (2011) observed an influence of spontaneous interpersonal coordination on postural transition frequency. However, they did not document whether it benefited the intrapersonal level. In these two studies, intrapersonal coordination α_intra_ was intentional and strong, and interpersonal coordination β_inter_ was spontaneous and weak. Based on our model, this led to a weak transfer at the intrapersonal level and no or little beneficial effect in terms of intrapersonal coordination stability. Conversely, our results echo those from Ramenzoni et al. (2012) showing that a strong interpersonal coupling is needed to observe a beneficial effect on spontaneous intrapersonal coordination and its stability. However, it seems that this beneficial effect is restricted to the instructed ML direction. Indeed, we observed that performing synchronized and improvised arm movements disturbed IPC in the AP direction. The given instructions did not require participants to coordinate their end-effector movements in this direction leading to a weaker interpersonal coupling (β_inter_). The transfer on the intrapersonal level is therefore too weak to positively impact coordination (α_intra_) and counterbalance the negative effect of complexity of improvised movements on IPC. Finally, the end-effector coordination required in the ML direction could also yield the emergence of bodily coordination between the two interactants (Schmidt et al., 2014). This bodily coordination could reinforce the positive effect of the interpersonal coordination in this direction. It therefore suggests that bodily interpersonal coordination could promote IPC stability in general, and not only body sway stability (Varlet et al., 2014).

Altogether, our results show that interpersonal coordination influences differently our postural stability in both directions. It promotes IPC stability in the instructed direction but disturbs simultaneously its stability in the uninstructed direction. Balasubramaniam et al. (2000) showed that a greater difficulty in a manual-aiming task was associated with reduced postural activity in one direction and with a simultaneously increased postural activity in the orthogonal direction. In the same vein, our results suggest a possible co-evolution between postural activities in both directions, with a decrease in stability in one direction accompanying by an increase in stability in the other.

### Social Roles Modulate IPC

Whereas we expected the follower role to induce more stability in IPC, we only observed a lower occurrence of antiphase pattern in the AP direction for the follower compared to the leader, suggesting that being a follower disturbs more IPC than being a leader in the uninstructed direction. This result echoes Ramenzoni et al.’s (2012) results showing that social role during the interaction differently modulates postural coordination. Although both participants produced synchronized and quasi-similar end-effector movements, the follower exhibited a typical signature of followership at the level of the end-effector – the *jitter* (Noy et al., 2011, 2015; Gueugnon et al., 2016). Jitter refers to a supplementary high frequency movement component (around 1.5 Hz) and witnesses the incertitude related to the follower role in which the participant has to imitate and mirror leader’s movements (Leader: 0.07 and Follower: 0.10). As the beneficial effect of interpersonal coupling is absent in AP, this incertitude could disturb postural stability yielding a decrease in the occurrence of stable pattern of the follower compared to the leader.

In the ML direction, this negative jitter effect was probably counterbalanced by the positive effect of interpersonal coupling previously described. In the Duo situation, both participants intentionally coordinated their end-effector movements leading to a strong coupling in the instructed direction. Coupling directionality refers to the influence of one oscillator onto the other (Schmidt and Richardson, 2008). In this particular situation, coupling is not symmetrical since the follower tracks the leader’s movements more than the leader tracks the follower’s movements. The follower being more influenced by the leader, the effect of such a coupling could be more powerful. As interpersonal coordination has a beneficial effect on IPC in ML, this effect should be reinforced for the follower yielding an improvement of IPC stability compared to the leader. However, this potential increase in the follower’s IPC stability could have been counterbalanced by the jitter present in the follower’s movements contributing to a reduction of the postural stability. In short, these positive and negative effects were probably compensating each other in the ML direction.

Obviously, investigating IPC in such an improvisation task has some limits. We interpret the current results as an influence of interpersonal coordination on postural stability, in line with other recent studies (e.g., Ramenzoni et al., 2012; Varlet et al., 2014). However, in Solo and Duo situations participants did not perform exactly the same movement, and it is not unreasonable to think that movements performed in Duo situations required more postural stability than those performed in Solo situations. Further studies will be required to address this issue specifically, for instance by asking participants to perform the exact same movements in both situations. To do so, a possibility would be to learn the movement produced in the Duo situation and to reproduce it in the Solo situation, but this would bring forward other limits related to movement acquisition. Although the current protocol is not ideal, we believe it is an optimal compromise to investigate the effect of movement creativity and of social coordination on postural stability. Finally, although a large number of subjects was included in the analysis, only one trial per condition and per included participant was used in our analysis. Therefore, we have excluded 22% of participants due to a loss of markers inherent to our experimental paradigm. Future work should evaluate the intra-individual consistency of these results through more repetitions.

## Conclusion

In this study, we aimed to investigate the IPC underlying social interactions (i.e., socio-motor improvisation), and to identify how creativity of arm movements and interpersonal coordination constrain posture. We demonstrated that stable IPC emerged during unilateral arm movements. In the instructed direction, an in-phase postural coordination spontaneously emerged to comply with the task instructions and facilitate end-effector movements; in the uninstructed direction, an antiphase pattern was adopted to maintain a stable proximal support. Whereas complexity of arm movements decreased IPC stability in both directions when there was no social interaction, interpersonal coordination counterbalanced this effect by promoting IPC stability in the instructed direction. In other words, performing improvised and synchronized end-effector movements induced a higher postural stability to facilitate these movements (in the instructed direction), together with a decrease in the stability of the proximal support in the orthogonal direction. This loss of stability was more important for the follower. We believe that our findings provide a more exhaustive overview of postural coordination in natural social situations, and contribute to a better understanding of our daily behaviors in which posture plays a fundamental role. Future research could also exploit these findings for postural rehabilitation in clinical contexts by adding interpersonal motor tasks that promote postural stability.

## Author Contributions

MG, RS, BB and LM designed the experiment; MG, AC and ZZ performed the experiments; MG, RS, BB and LM analyzed the data; and MG, RS, BB and LM wrote the manuscript.

## Conflict of Interest Statement

The authors declare that the research was conducted in the absence of any commercial or financial relationships that could be construed as a potential conflict of interest.
